# Moving Barbados forward: a case for investment in exercise for geriatric health

**DOI:** 10.3389/fpubh.2026.1926810

**Published:** 2026-08-12

**Authors:** Sanjay Seegobin, Cordelle Lazare, Leslie S. Craig, Sheldon Jones, Rhonda McIntyre

**Affiliations:** 1 Ross University School of Medicine, Bridgetown, Barbados; 2 Healthier Nation Initiative, Bridgetown, Barbados

**Keywords:** Barbados, chronic noncommunicable diseases, community-based exercise program, fall prevention, geriatric health, health policy, healthy aging, physical activity

## Abstract

Barbados faces a geriatric health crisis: by 2060, older adults will comprise 29.1% of the population; NCDs cause 83% of deaths, and adults are inactive. While citizen group exercise programs exist, no government-funded, community-based geriatric exercise program operates at the national scale in Barbados or across the wider Caribbean region. Evidence shows exercise programs reduce fall risk and generate 36 to 127% return on investment, with the greatest financial benefit accruing for the oldest and highest risk patients. A government partnership with Healthier Nation Initiative Foundation, using existing polyclinic infrastructure, offers a cost-effective, scalable, evidence-based preventive strategy. A phased rollout is recommended, starting with Active Agers pilot in Drax Hall, with clinical monitoring supported by relevant stakeholders.

## Introduction

Barbados is experiencing a rapid demographic shift toward an aging population that is placing an unsustainable economic and clinical burden on the national healthcare system (1). Barbados operates a centralized, nationally administered healthcare system under the jurisdiction of the Ministry of Health and Wellness, in which non-communicable diseases (NCDs) account for 83% of all deaths nationally and consumes approximately 70% of the Queen Elizabeth Hospital’s (QEH) total annual operating budget (2–4). Their impact is disproportionately concentrated among older adults, with an estimated 75% of deaths in persons aged 65 years and older attributable to NCDs (2–4). Although age-standardized mortality rates have improved, deaths driven by population aging has increased by 35%, highlighting aging as a major determinant of the NCD burden (5). This demographic shift is reflected in the growing proportion of older adults, now 16.6% of the population. The economic implications differ significantly from the general population: while national estimates highlight substantial annual expenditures (e.g., BBD $64 million on cardiovascular disease and diabetes and BBD $145 million in productivity losses), the older adult population generates predominantly long-term, system-intensive costs (6). These include prolonged hospitalizations and institutional care, with “abandoned” older adult patients alone costing approximately BDS $25,000 per month per individual (7). Such costs reflect the downstream consequences of poorly controlled NCDs (functional decline, frailty, and dependency) and place a disproportionate burden on hospital resources, accounting for substantial utilization of the healthcare infrastructure.

Physical inactivity is a primary driver of this crisis. Data indicates that physical inactivity affects 43% of Barbadian adults serving as a root cause of obesity, hypertension, functional decline, and geriatric falls (1). Falls are not merely a symptom of aging but a direct, preventable consequence of inactivity. More than 26% of geriatric patients in Barbados experience at least one fall annually, often resulting in premature death or permanent loss of independence (8). The cost of managing this physical and medical decline is substantial. Inadequately managed NCDs and progressive functional decline drive seniors toward dependence on acute care beds, placing pressure on hospital resources that could be relieved through adequate community-level prevention. As the older adult population is projected to nearly double by 2060, representing 29.1% of the total population, the scale of unmet preventative need is only growing (1).

Despite these pressures, no government-funded community exercise program targeting the geriatric population exists on a national scale. This policy brief evaluates the evidence basis for community geriatric exercise as a preventive intervention, identifies the gap in Barbados’ current policy framework, and recommends a phased government partnership with the Healthier Nation Initiative (HNI) Foundation Inc. as the mechanism for closing that gap.

## Policy options and implications

### Current policy landscape and identified gaps

The Government of Barbados has made meaningful investments in older adult care infrastructure. The National Policy on Ageing (2023–2028) and the Alternative Care of the Elderly Program (ACEP) received BDS $38.3 million in the 2022/2023 fiscal year (9). The Government has also broken ground on a new purpose-built Geriatric Hospital at Waterford, St. Michael, which will provide 300 beds in its first phase and 408 beds upon full completion, representing the most significant investment in geriatric health infrastructure since the opening of the Queen Elizabeth Hospital (10). Additionally, the government recently approved BDS $4 million in financing for the construction of a new adult day program and respite facility in St. Philip (2, 3).

These investments, though essential, remain predominantly reactive and oriented toward managing functional decline rather than preventing it. The existing infrastructure is predominantly curative, comprising hospital beds and respite placements, while residential care exists as a distinct, non-curative component of the care system. While critical for acute and complex cases, these services are resource-intensive and do not address the upstream behavioral and physiological risk factors that drive preventable admissions. Research across Jamaica, Guyana, and Dominica confirm that community-based NCD prevention is highly effective but cannot achieve sustainability without formal policy and financial backing (11). The absence of an institutionalized, publicly funded physical activity framework within primary care represents a critical gap in the national health strategy (4). Closing this gap requires a deliberate policy shift from reactive care toward structured prevention delivered at the community level, embedded within Barbados’s existing primary care infrastructure.

### Evidence basis for community geriatric exercise

The evidence for structured physical activity as a geriatric health intervention is robust and consistent across settings. Comprehensive meta-analyses of over 100 randomized controlled trials demonstrate that balance and functional training reduce fall rates by at least 23% (12). Systematic reviews have shown that participation in fall prevention programs, namely the Otago Exercise Program, reduces fall risk while improving balance, gait, and lower limb strength, although the magnitude of the benefit varies across settings (13, 14). Regular physical activity reduces cardiovascular mortality by 39–50% in older adult cohorts and is associated with improved cognitive function, reduced depression, and delayed entry into institutional care (15, 16).

Economic evaluations of fall prevention exercise programs reinforce these findings at the health system level. A cost benefit analysis of the Otago Exercise Program found a net benefit of $121.75 USD per participant and a 36% return on investment for adults aged 65 and older, raising a net benefit of $429.08 USD and a 127% return on investment for those aged 80 and older (17). A 2022 systematic review of 24 economic evaluations further found that most fall prevention exercise programs were cost-effective, with the greatest value accruing for the older adults at high fall risk (18). Critically, these benefits are not confined to high-income settings. Evidence from low- and middle-income countries demonstrates that group-based exercise programs, when delivered through community infrastructure and supported by trained facilitators, yield comparable outcomes at significantly lower cost (19). The transferability of these models to small island developing states like Barbados is supported by shared characteristics: high NCD prevalence, aging demographics, and health systems under fiscal pressure. In this context, the cost-effectiveness argument for prevention is not just theoretical; it is an operational necessity.

### Proposed policy option: government–HNI partnership

The recommended policy option is a formal government partnership with HNI Foundation Inc., channeled through the Ministry of Health and Wellness and the Ministry of People Empowerment and Elder Affairs. Specifically, the government’s role would be to support the scale-up and integration of HNI’s Active Agers program within existing public health delivery platforms, including polyclinics and community sites, while establishing and maintaining a formal collaboration with HNI for program implementation and oversight. Structured geriatric exercise classes would be made available at no cost to eligible seniors through these platforms. Eligible seniors would be identified through polyclinic-based screening, HNI/Ross University School of Medicine collaborative pop-up clinics, general practitioner referral, and community outreach, ensuring equitable representation across all 11 parishes.

HNI, established in 2020 and currently operating in Barbados, Grenada, and Antigua and Barbuda, delivers affordable wellness solutions across the Caribbean (20). Central to this proposal is HNI’s Active Agers program, a structured group exercise intervention designed specifically for the geriatric population and currently being piloted in Drax Hall, Barbados. The Active Agers program delivers supervised strength, balance, and mobility training targeting the physical risk factors associated with falls, cardiovascular decline, and functional loss. The program is tailored for the geriatric population, addressing the precise physiological deficits such as reduced muscle strength, impaired balance, and diminished gait speed, that underlies preventable falls and functional dependence.

HNI also operates the “21-for-21” program, providing 21 min of daily structured exercise accessible via the organization’s website.^1^ This multimodal delivery model ensures participation by rural and mobility-impaired populations. The HNI model is explicitly designed for equity. Remote delivery addresses rural parishes with limited transportation access, ensuring the program benefits are not concentrated among those who are already advantaged. Because HNI is already operational across three Caribbean territories, this proposal represents a strategic scale-up of an established organization rather than a high-risk start-up. By formally adopting this model, Barbados has an opportunity to become a regional leader in evidence-based geriatric wellness and establish a replicable standard for the entire CARICOM community.

This investment is projected to yield a positive financial return through reductions in fall-related hospitalizations and the number of seniors requiring government-subsidized care. Published cost–benefit analyses demonstrate that structured exercise programs for older adults generate net positive returns on investment, with returns increasing substantially for the older, higher risk participants (17, 18). Beyond falls, measurable improvements are expected in blood pressure, body mass index (BMI), HbA1c, and overall quality of life, directly targeting the NCD risk factors driving Barbados’s healthcare burden (21).

Clinical quality assurance and program evaluation will be conducted in partnership with academia. Outcome measures will include fall rate, fall-related hospitalizations, blood pressure, BMI, balance scale assessment, gait speed, grip strength, HbA1c, and validated quality-of-life instruments.

### Implementation considerations

Implementation should follow a phased approach. Phase 1 formalizes the existing Active Agers pilot in Drax Hall as the official national pilot, generating a real-world evidence base from which delivery protocols can be refined before national expansion. The program should be integrated within existing service structures: ACEP, polyclinics, and community social services, to minimize duplication, reduce administrative burden, and ensure clear referral pathways.

Key stakeholders for engagement include:

- Ministry of Health and Wellness (policy oversight and funding authorization)
- Ministry of People Empowerment and Elder Affairs (policy oversight and funding authorization)
- Ministry of Industry, Innovation, Science and Technology (policy oversight for internet access)
- Barbados National Commission on Chronic Non-Communicable Diseases (technical guidance and NCD strategy alignment)
- Transport Board (senior transportation logistics)
- National Assistance Board (participant identification and referral)
- HNI Foundation Inc. (program delivery and instructor training)
- Academia (clinical monitoring, quality assurance and data evaluation)

Barriers including senior transportation and instructor certification will be mitigated by leveraging HNI’s established training protocols and digital broadcast capabilities. Long-term participant retention will be supported by prioritizing public venues; in-person group settings provide the social engagement that serves as a powerful motivational driver for older adults.

## Actionable recommendations

- The Government of Barbados should formalize a partnership with HNI Foundation Inc. through the Ministry of Health and Wellness and the Ministry of People Empowerment and Elder Affairs to support the scale-up and integration of community geriatric exercise programming within existing public health delivery platforms. This formalized commitment is expected to establish the institutional foundation required for sustainable, nationwide program delivery, providing seniors with structured access to preventive physical activity services through the polyclinic network.
- The Active Agers pilot program in Drax Hall should be formally designated as Phase 1 of the national rollout, with standardized protocols, defined outcome metrics, and a clear expansion timeline tied to demonstrable results. This designation will generate a real-world evidence base under Barbadian conditions, enabling data-driven refinement of delivery protocols before national scale-up, thereby reducing implementation risk and strengthening the program’s credibility with policymakers and the public. This could be piloted via a phased approach, starting in one central polyclinic and expanding to additional polyclinics based on implementation experiences and feasibility.
- The partnership model should be determined in consultation with relevant ministries and formalized through appropriate legislative or administrative mechanisms. Establishing a clear legal and administrative framework will ensure accountability, define the respective roles of government and HNI, and provide a durable structure that protects program continuity across changes in government or institutional leadership.
- Funding should be embedded within the existing ACEP budget framework and aligned with the National Policy on Ageing (2023–2028) to ensure institutional continuity beyond electoral cycles. Budget integration within the ACEP framework will protect the program from funding interruptions, signal long-term government commitment, and position the intervention as a core component of Barbados’s older adult care strategy rather than a temporary initiative.
- A monitoring and evaluation framework should be established from program inception, including the development of data collection plans and the clear assignment of organizational responsibilities for data collection. Standardized reporting of key outcomes such as fall rates, fall-related hospitalizations, NCD biomarkers, and quality of life should be conducted on a biannual basis. Early and consistent data collection will enable evidence-based program refinement, demonstrate return on investment to government stakeholders, and contribute to the growing regional evidence base for community geriatric exercise across CARICOM.
- The Transport Board should be engaged as a formal delivery partner to address transportation barriers for seniors in rural parishes, with HNI’s radio and television broadcast capability deployed as a supplementary access mechanism. Addressing transportation as a structural barrier to participation is expected to significantly increase program reach among rural and mobility-impaired seniors, ensuring that the program delivers equitable health benefits across all parishes and does not disproportionately serve those with pre-existing access advantages.

## Conclusion

Barbados faces three compounding public health challenges: a rapidly aging population, an NCD burden that is among the highest in the Caribbean, and a critical gap in preventive geriatric care. The evidence clearly supports structured community exercise as an effective, cost-efficient intervention capable of reducing falls, improving NCD biomarkers including BMI, blood pressure and HbA1c, delaying institutionalization. Without a shift toward integrated, preventive exercise programming, the financial demands of reactive geriatric care will continue to grow, placing unsustainable pressure on national resources. A government partnership with HNI Foundation Inc. offers a proven, scalable, and equitable mechanism for delivering this intervention at a national scale, transforming the aging experience from one of managed decline to one of active wellness. The evidence-based results of this initiative prove that functional loss does not have to be an inevitable part of aging. The time to act is now to ensure a healthier, more resilient future for the seniors of Barbados.

## Footnotes

1
https://hnicaribbean.com/
